# Synergistic Effect of Partial Replacement of Carbon Black by Palm Kernel Shell Biochar in Carboxylated Nitrile Butadiene Rubber Composites

**DOI:** 10.3390/polym15040943

**Published:** 2023-02-14

**Authors:** Zafirah Zainal Abidin, Siti Nur Liyana Mamauod, Ahmad Zafir Romli, Siti Salina Sarkawi, Nahrul Hayawin Zainal

**Affiliations:** 1 Faculty of Applied Sciences, Universiti Teknologi MARA, Shah Alam 40450, Selangor, Malaysia; 2 Centre of Chemical Synthesis and Polymer Technology (CCSPT), Institute of Science, Universiti Teknologi MARA, Shah Alam 40450, Selangor, Malaysia; 3 Technology and Engineering Division, Malaysian Rubber Board, RRIM Research Station, Sungai Buloh 47000, Selangor, Malaysia; 4 Biomass Technology Unit, Malaysian Palm Oil Board (MPOB), Kajang 43000, Selangor, Malaysia

**Keywords:** carbon black (CB), palm kernel shell biochar (PKSBc), carboxylated nitrile butadiene rubber (XNBR), rheological properties, abrasion resistance, hardness

## Abstract

With the rapid development of the palm oil-related industry, this has resulted in the high production of palm oil waste. The increasing amount of palm oil waste has become an alarming issue in which researchers have carried out studies that this palm oil waste has the potential to be used as a biomass source. Carbon black (CB) is the most preferred reinforcing filler in the rubber industry but it has a disadvantage where CB is carcinogenic and a petroleum-based product. Hence CB is less sustainable. Palm kernel shell (PKS) derived from palm oil waste can be turned into palm kernel shell biochar (PKSBc) which can potentially be a value-added, sustainable biofiller as reinforcement in rubber composites. In this study, PKSBc is hybridized with CB (N660) at different loading ratios to be filled in carboxylated nitrile butadiene rubber (XNBR). This study aims to elucidate the effect of the varying ratios of hybrid CB/PKSBc on the rheological properties, abrasion resistance, and hardness of XNBR composites. In this study, both CB and PKSBc are incorporated into XNBR and were then cured with sulphur. The composites were prepared by using a two-roll mill. Different compositions of hybrid CB/PKSBc were incorporated. The rheological properties and physicomechanical properties, such as abrasion resistance and hardness of the vulcanizates, were investigated. Based on the results, as the loading ratio of PKSBc in hybrid CB/PKSBc increases, the cure time decreases, and the cure rate index increases. The abrasion resistance and hardness values of vulcanizates were maintained by the high loading of PKSBc which was due to the porous structure of PKSBc as shown in the morphological analysis of PKSBc. The pores of PKSBc provided mechanical interlocking to reduce volume loss and maintain the hardness of vulcanizates when subjected to force. With this, PKSBc is proven to be a semi-reinforcing filler that could not only act as a co-filler to existing commercialized CB, but PKSBc could also fully substitute CB as reinforcement in rubber, specifically XNBR as it is able to provide high abrasion resistance and hardness to the rubber composites. This would mean the performance of PKSBc is comparable with CB (N660) when it comes to maintaining the physicomechanical properties of XNBR composites in terms of abrasion resistance and hardness. Therefore, this approach of using eco-friendly filler derived from palm oil agricultural waste (PKSBc) can reduce the abundance of palm oil waste, be a sustainable alternative to act as a co-filler in hybrid CB/PKSBc to decrease the usage of CB, and helps to enhance the quality of existing rubber-based products.

## 1. Introduction

Filler is one of the compounding ingredients that is needed in rubber compounding. There are many filler types available in the rubber industry and they can either be organic or inorganic [1]. Carbon black, clay, calcium carbonate, and calcium silicate are examples of fillers. When a filler is added it helps to increase the modulus, abrasion, and tear resistance of the rubber [2]. In the rubber industry, carbon black (CB) plays an important role as a reinforcing filler [3,4]. CB is widely used and the dominant filler in the rubber industry because of its reduced particle size and has a huge range of grades [5]. However, CB needs to be substituted with renewable filler due to some negative adverse and disadvantages [6]. CB is known to be a petroleum-based product, hence it is not renewable and sustainable [7]. This is because CB is produced by the partial combustion or thermal decomposition of petroleum hydrocarbons and it has a considerable carbon footprint [8]. Moreover, CB is carcinogenic which can cause difficulty in breathing and even cancer too if high and long-term exposure to CB is involved [9]. These reasons have then encouraged researchers to find other solutions which could reduce the utilization of CB in the rubber industry. As the awareness of the environment and future availability of rubber resources has been increasing, it has become important that other methods and natural materials from renewable, sustainable, and environmentally friendly sources are used [10]. There have been many studies carried out to get the best solution to benefit from natural, renewable resources to overcome pollution issues, encourage a sustainable way of living, and shift from non-renewable petroleum-based materials to more sustainable renewable resources which eventually became high in demand by the society [11,12].

In the rubber industry, the utilization of natural fillers from natural resources is very beneficial as it lowers the costs of production as cheaper materials (natural fillers) are used [11]. Natural fillers are known to be environmentally friendly, fully degradable, sustainable, available in abundance, cheap, and low in density [9,13,14]. In the last few years, sustainable materials derived from plants, such as cellulose nanocrystals, natural fibers, lignin, and biochar are widely incorporated in various studies as a replacement or complementary fillers for rubber composites [8]. There are a few choices of natural fillers and filler resources available in Malaysia, such as bamboo, rattan, pineapple leaves, wood flour, starch, betel, and kenaf. Palm oil is also one of the resources for natural filler too. There has been some research conducted to investigate the potential of palm oil waste to be turned into natural fillers. Since there is an abundance of palm oil waste that is discarded all over the world, this waste could be utilized to its fullest potential by converting them into biofillers. Palm oil waste especially PKS could be a possible source for cellulose-based natural fibers and particles. It can be said that PKS is a good source of biomaterials. However, PKS has a few disadvantages, such as high moisture content and low carbon content. Since PKS is a lignocellulosic material, the properties of rubber composites filled with PKS are expected to be lower compared to those filled with CB [7]. PKS also contains contaminants such as oil sludge and fungus. In order to overcome this problem, PKS undergoes a pyrolysis process to remove these contaminants, reduce the moisture content and increase the carbon content of the PKS where the final product is known as PKSBc. The process of synthesizing biochar via pyrolysis is becoming popular to avoid unsustainable and non-renewable petroleum supply chains [15]. This aids in achieving sustainability as the products of pyrolysis can be recovered for a wide range of applications, which, in this study, PKSBc can be used as filler to provide reinforcement in the rubber and polymer industry. This pyrolysis byproduct, PKSBc has been getting recognition due to emerging research on its implementation for composite materials to provide an alternative to CB which is petroleum-based. Higher sustainability can be achieved with the usage of PKSBc. The addition of natural-based fillers into the rubber compounds is a very effective and economical alternative to meet the needed requirements for specific applications. This way, by determining the effects of PKSBc in rubber composites, the possibility of PKSBc as an alternative filler to either completely or partially replace the usage of CB in rubber compounding can be studied. Utilization of PKSBc from palm oil waste can contribute to the economy and nature as it reduces waste, protects our environment, and recycles them for other sorts of applications. The mechanical properties of the composites can be further enhanced by the excellent stiffness of these fillers [16]. This alternative would bring many benefits to many countries especially our own to a more eco-friendly, sustainable, and bright future. Innovation in producing biochar from PKS provides an alternative to decreasing the usage of CB which is petroleum-based, in rubber products. Therefore, providing a solution to reduce the abundance of palm oil waste. This can enable the efficient use of natural resources from palm oil waste especially, to help with the transition towards a low-carbon economy and invest in sustainable solutions where PKSBc is being utilized and CB from fossil fuels is reduced. Although PKSBc as a filler in thermoplastic was reported by a few previous researchers, there are limited studies involving the usage of PKSBc as a filler in rubber-filled vulcanizates [17].

In this research, XNBR composites are filled with hybrid CB/PKSBc at different loadings where XNBR acts as the main rubber component, CB as the reinforcing filler, and PKSBc as the natural biofiller derived from palm oil waste. The main idea of this research is to study the synergistic effect between CB and PKSBc as fillers in rubber. This research aims to study the rheological and physicomechanical properties of the final composite. This way the effectiveness of the hybridization of CB with PKSBc can be proven to further enhance the mechanical properties, hence improving the rubber composite’s performance, and at the same time provide an effective, sustainable alternative to overcome palm oil waste abundance and reduce the usage of CB. With this, PKSBc could be commercialized as a semi-reinforcing filler and utilized by rubber manufacturers as an alternative to CB in rubber compounding. This study will also encourage rubber researchers and manufacturers in the rubber industry to utilize PKSBc in the production of rubber-based products in various applications. For instance, the transportation and footwear industry.

## 2. Materials and Methods

This study consists of two parts, Part A and Part B. Part A is where the PKSBc is prepared from palm oil waste and characterized through Fourier transform infrared (FTIR) analysis and scanning electron microscope (SEM) analysis while Part B is the preparation of hybrid CB/PKSBc filled XNBR composite and characterization of the composites through rheological, abrasion resistance and hardness tests.

### 2.1. Materials

The materials used in this study were XNBR, PKSBc, CB, zinc oxide (ZnO), stearic acid (HST), benzothiazyl disulfide (MBTS), dipropylene glycol (DPG), tetramethylthiuram disulfide (TMTD), polymerized 2, 2.4-trimethyl-1.2-dihydroquinoline (TMQ), sulphur, and processing oil (paraffin oil). The XNBR used in this study was Krynac X 750 (7 wt.% carboxyl group content, 27 wt.% nitrile contents). XNBR was supplied by a company named Aras Bakti Ventures Sdn. Bhd. Compounding ingredients such as MBTS, DPG and TMQ were supplied by a local company named Airelastic Industries Sdn. Bhd. CB and PKSBc were both used as a filler for XNBR.

### 2.2. Methods

#### 2.2.1. Part A: Preparation of Fine Particles PKSBc

The PKSBc was obtained from the Research Institute of Malaysian Palm Oil Board (MPOB) Bangi, Malaysia and they were in big particle size. Firstly, the PKSBc was crushed by using a crusher. The initial PKSBc obtained from MPOB were then transformed into smaller pieces. These small pieces of PKSBc were then pulverized with a pulverizer and sieved through a sieve, with a mesh size of 240 (equivalent to 53 µm), using a vibratory sieve shaker (Retsch AS 200, Haan, Germany).

#### 2.2.2. Part A: Characterization of PKSBc

FTIR Analysis. The surface chemical properties of palm kernel shell-based biochar were studied through Fourier transform infrared (FTIR) spectroscopy (Perkin Elmer, Waltham, MA, USA), carried out to determine the chemical functional groups present in the PKSBc. A thin KBr (potassium bromide) disc method was used for this FTIR analysis. Tools were cleaned with acetone to get rid of any moisture present on the surface that could disturb the FTIR outcome. The data for FTIR was collected with a range of 4000 to 650 cm^−1^ wavelength and a resolution of 4 cm^−1^.

SEM Analysis. Morphological characteristics of the palm kernel shell biochar samples were determined by using a scanning electron microscope called Shimadzu SSX-550 Superscan Scanning Electron Microscope (Shimadzu, Kyoto, Japan). Each sample was placed in the holder of SEM and a layer of gold with about 20 nm thickness was sputter coated on the samples before any observation was carried out. The coating helped to prevent any electrostatic charging and give a better image resolution. With SEM analysis, the appearance or structure morphology of the palm kernel shell biochar samples could be studied through the SEM micrographs.

#### 2.2.3. Part B: Preparation of Hybrid CB/PKSBc Filled XNBR Composites

The formulation of hybrid CB/PKSBc-filled XNBR composites where the CB and PKSBc were incorporated in the XNBR compounds in various proportions is summarized in Table 1 below. Before the process of vulcanization, ZnO, stearic acid, MBTS, DPG, TMTD, TMQ, sulphur, and processing oil were incorporated into each compound. The mixing process was carried out by using a two-roll mill, in accordance with the American Standard of Testing and Material (ASTM) designation D3184-80. The compounded rubber was then placed in a mould and pressed between the heated platens of a hydraulic press at 160 °C to be cured at a specific time based on the rheological properties results. After that, abrasion resistance and hardness tests were conducted.

#### 2.2.4. Part B: Testing and Characterization of Hybrid CB/PKSBc Filled XNBR Composites

Rheological properties test. A rheometer test was conducted before curing the blends in the press, to evaluate whether it was needed to carry out a readjustment of the prepared formulation. The rheological properties were studied at 160 °C using a Monsanto Moving Die Rheometer with about 4g of sample of the respective compounds. Data obtained through the rheological test include minimum torque (M_L_), maximum torque (M_H_), delta torque (M_H_–M_L_), scorch time (T_S2_), optimum cure time (T_90_), and cure rate index (CRI).

Abrasion resistance test. Abrasion resistance plays an important role in determining the performance of the final product. The abrasion test is able to foresee the total stability and strength of the rubber when performing for specific applications, especially those requiring abrasion resistance. Rotatory Drum Abrasion which is also known as the DIN Abrasion test was carried out to characterize the XNBR composites. The test was carried out in accordance with ISO 4649 by using the DIN abrasion resistance tester. This method evaluated the volume change because of the friction force contributed by the abrasive action. The obtained abrasion results were recorded as a relative volume loss.

Hardness test. The material’s hardness is defined as the resistance from being permanently deformed or indented. The hardness test was carried out according to ASTM D 2240 method and analysed by a Durometer (shore type A). Generally, in the hardness test, the surface of the rubber sample was subjected to a hard indenter and the indenter was pressed deeply onto it, where a fixed load was applied for an amount of time. The measurement of hardness value was taken based on the indention’s size or depth. The hardness value was taken after a contact of about 15 s with the Shore A indenter which was obtained at three different points on the test piece.

## 3. Results and Discussion

### 3.1. Part A: Characterization of Palm Kernel Shell Biochar (PKSBc)

#### 3.1.1. FTIR Analysis

**Figure 1:**
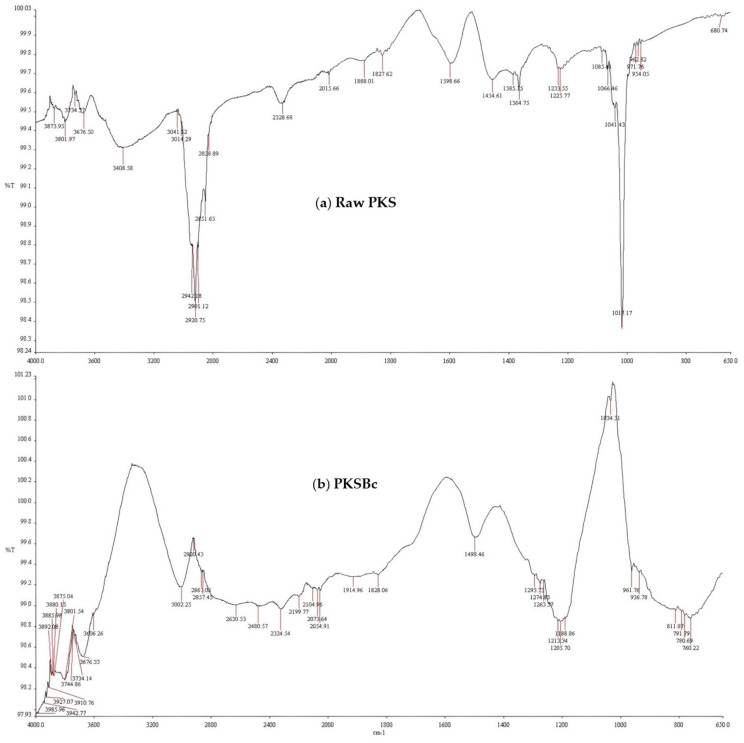
FTIR spectrum comparison between (**a**) raw palm kernel shell and (**b**) palm kernel shell biochar.

Figure 1a,b shows the FTIR spectrum of raw palm kernel shell and palm kernel shell biochar respectively. It clearly shows that the biochar displayed a distinctive FTIR spectrum compared to the raw biomass. The broad absorption bands at approximately 3600 cm^−1^ showed by palm kernel shell biochar corresponded to a sharply decreased intensity of the O-H stretching of hemicellulose and cellulose which had diminished. The decomposition and volatilization of cellulose and lignin components contributed to the results of the FTIR spectrum. At about 2920 cm^−1^ which represents the C-H stretching of cellulose, hemicellulose had degraded entirely which indicates the decompositions of the involved components during the process of pyrolysis. Since the pyrolysis temperature of the PKSBc is at 500 °C, functional groups such as hydroxyl, which is initially and weakly linked on the main chain of the main components (cellulose, hemicellulose, and lignin) in the raw PKS, would steadily reduce under thermal cracking. Pyrolysis of the raw PKS promotes the formation of oxygen-containing functional groups such as alcohols, phenols, and carboxylic acids [18]. The appearance of aromatic C=C stretching (1490 cm^−1^–1700 cm^−1^) displayed an increment in the degree of condensation and aromatization of the palm kernel shell biochar organic compound. Similar spectrum results were reported in previous studies involving palm kernel shells [19,20] and other lignocellulosic materials such as biomass of pinewood [21] and walnut shells [22].

#### 3.1.2. SEM Analysis

**Figure 2:**
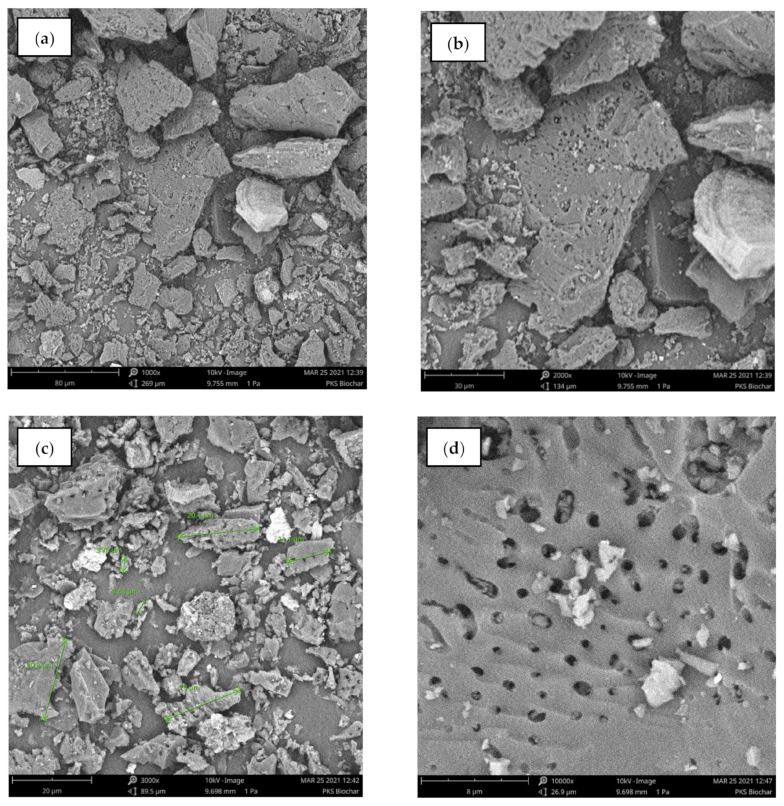
SEM images of PKSBc at different magnifications (**a**) ×1000, (**b**) ×2000, (**c**) ×3000, (**d**) ×10,000 which show the porous structure of PKSBc.

**Figure 3:**
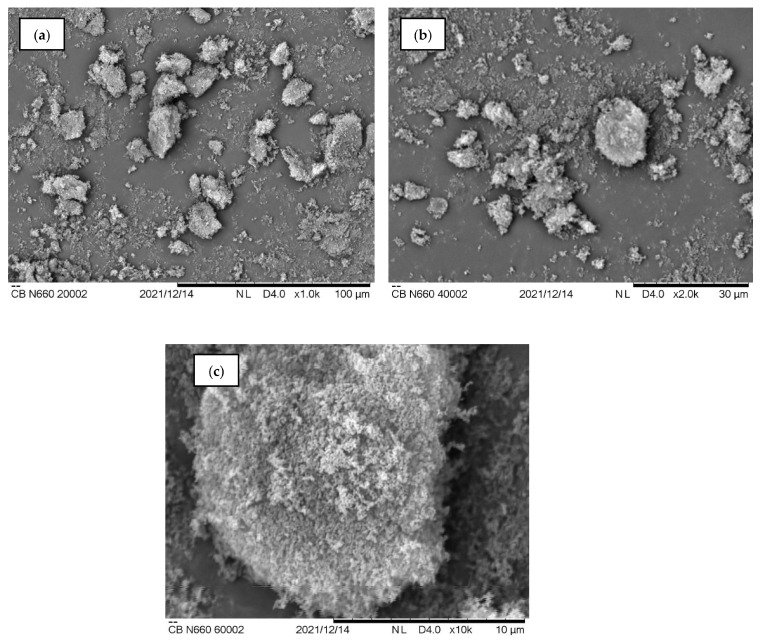
SEM images of CB at different magnifications (**a**) ×1000, (**b**) ×2000, (**c**) ×10,000 show the regular shape and smooth surface of CB particles.

Figure 2 illustrates the SEM analysis of the palm kernel shell biochar obtained in this study. For comparison reasons, the same magnification scale is applied for both CB and PKSBc during the SEM imagining analysis. Raw palm kernel shell (PKS) has been proven to be non-porous as the surface is flat and rather compactly filled [23,24,25]. Moreover, no apparent cracks or crevices can be observed on the surface. Impurities were visible on the surface which could be contributed by the availability of contaminations such as fungus, insects, and sludge present in the raw PKS structure. However, once the raw PKS underwent pyrolysis, the high temperature applied resulted in a significant transformation on the surface morphology of the biochar. The impurities and volatile matter will then evaporate with the increased temperature during pyrolysis, hence creating pores and increasing the surface area of the PKSBc [26]. The release of volatiles was resultant of the biomass particles being exposed at high-temperature conditions. This is in accordance with the proximate analysis of the raw PKS and PKSBc, where once the raw PKS undergo the pyrolysis process, the volatile matter in the raw PKS had a reduction from 53.4 wt.% to 13.1 wt.%. The SEM images shown in Figure 2a,b depicts irregular-sized biocarbon particles (refer to the red arrows), and that the particle size of PKSBc is less than 35 μm size (refer to the red arrows in Figure 2c). The surface of the biochar displayed roughness with a sponge-like structure where it could be seen that there is a large number of pores, cavities, and cracks on the surface of the biochar as shown in Figure 2d. The pyrolysis process causes the PKSBc to have a lower density, higher porosities, and improved pore structure [27]. The developed porosity looked akin to circular shaped openings. Since the biochar is derived from a natural biomass source which is PKS, the lack of impurities such as tar which can cause the pores to be clogged, contributed to the good pore structure development. The structure of the biochar also showed some surface etchings and seemed to be brittle and breakable which is shown in Figure 2b, by referring to the blue arrows. The presence of pores on the surface of the biochar indicates the effectiveness of the pyrolysis process in producing palm kernel shell biochar with a porous surface. Figure 3 displays the SEM images of commercial carbon black (CB) of grade N660. The commercial CB seemed to have a smoother surface composed of a fine-grain structure. The N660 grade of carbon black certainly is finer when compared to the SEM images of PKSBc [28]. Moreover, the particle of CB is more regularly shaped than PKSBc which has an irregular shape. Surface morphology plays an important role in the reinforcement effect of any carbon black. The particles of the CB showed quasi-spherical and elongated shapes which helped to provide great interfacial and bigger contact area (refer to the red arrows shown in Figure 3a,b). It could also be observed that the morphology of carbon black had a smooth surface structure which can be seen in Figure 3c. Similar SEM images are reported by previous studies where the CB particles were regularly shaped and more rounded [29,30,31].

### 3.2. Part B: Characterization of Hybrid CB/PKSBc Filled XNBR Composites

#### 3.2.1. Rheological Properties of Hybrid CB/PKSBc Filled XNBR Composites

Table 2 shows the rheological properties of hybrid CB/PKSBc-filled XNBR composites. The data and curves were obtained from an MDR rheometer at 160°C. From the rheometer test, there were a few data obtained including M_L_, M_H_, delta torque (M_H_ − M_L_), T_S2_, T_90_, and cure rate index (CRI). Based on the results in Table 2, it can be seen that the value for unfilled XNBR composite is the lowest among all composites. In comparison with the unfilled XNBR composite, the incorporation of 35 phr of CB in the XNBR composite increased the ∆ torque values by approximately 32.7% while the addition of 35 phr of PKSBc in the XNBR composite caused ∆ torque values to rise by approximately 39.3%. The increased ∆ torque is an indication that the XNBR composites became hardened with the incorporation of hybrid CB and PKSBc filler. However, when a hybrid CB/PKSBc of 30/5 phr loading ratio is added into the XNBR matrix, the ∆ torque has a slight reduction compared to CB filled XNBR composite. The ∆ torque continued to decrease as the PKSBc loading increased in the hybrid CB/PKSBc. The decreasing ∆ torque of the hybrid CB/PKSBc-filled XNBR composites also indicated a reduction of crosslink density, hence lowering the modulus of XNBR composites [32]. The T_s2_ for unfilled XNBR was the highest. Unlike CB filled XNBR composite, the T_s2_ was reduced by 27.2% when compared to the unfilled XNBR composite. Hybrid CB/PKSBc filled XNBR composite at 30/5 phr loading ratio also showed shorter T_s2_ than unfilled XNBR composite but higher than CB filled XNBR composite. However, as the loading of PKSBc increased, the T_s2_ of the XNBR composites with hybrid CB/PKSBc decreased. With higher loading of PKSBc in the XNBR composite filled with hybrid CB/PKSBc, the T_90_ also showed a decreasing trend and is slightly lower than CB filled XNBR composite. This could be due to the surface activity of PKSBc where the PKSBc consists of hydroxyl groups (refer to FTIR analysis of PKSBc mentioned earlier). High surface activity of OH- groups resulted in the formation of filler-filler interaction where the presence of OH- groups on the surface of PKSBc cold accelerate the T_90_ [33,34]. As for the CRI of the XNBR composites, an increasing trend is observed with the addition of hybrid CB/PKSBc filler where the PKSBc composition is higher. Having high surface activity makes PKSBc highly polar and acts as a cure-activating compound in the XNBR matrix whereby increasing the loading ratio of PKSBc, the activating sites for vulcanization increase [33]. Therefore, the CRI of XNBR composites filled with a higher ratio of PKSBc increases. The CRI is where the crosslinking and development of the modulus of the compound occurs after the scorching point. It is known that the addition of filler into a rubber compound decreases the T_s2_ and T_90_. Therefore, PKSBc seemed to be an effective co-filler to be hybridized with CB as reinforcement for rubber compounding [28].

#### 3.2.2. Abrasion Resistance

**Figure 4:**
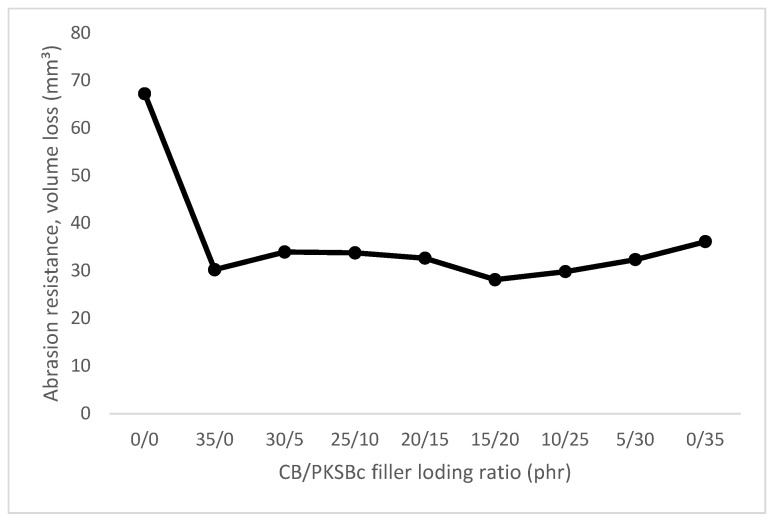
Volume loss of hybrid CB/PKSBc filled XNBR composites.

The incorporation of filler helps to promote reinforcement to the rubber composite which contributed to high abrasion resistance and low volume loss, which, in this study, the hybrid CB/PKSBc improved the abrasion resistance of the XNBR matrix. Figure 4 shows the results for volume loss of hybrid CB/PKSBc-filled XNBR composites. Based on the results in Figure 4, it can be observed that the unfilled XNBR composites had the highest volume loss compared to the filled XNBR composites. With no presence of filler, the composite had low mechanical resistance to resist surface damage. With the incorporation of 35 phr of CB into the XNBR matrix, it can be seen that the volume loss was decreased by more than half (54.9%). Reinforcement in filled rubber composites is highly affected by particle size, specific surface area, and structure of the filler. These characteristics would greatly influence the degree of reinforcement of rubber composites. Reinforcing filler such as CB has very fine particle size, active surface area, and porous structure to maximize rubber-filler interaction [11]. This is shown in the SEM images of CB in Figure 3a–c. Based on the SEM images, the particles of the CB were quasi-spherical and elongated in shape which increased the surface area of contact for rubber-filler interaction, hence helping to reduce volume loss of the XNBR composite, indicating high abrasion resistance. As for XNBR composites with hybrid CB/PKSBc with 30/5 phr, 25/10 phr, 20/15 phr, 15/20 phr, 10/25 phr, and 5/30 phr filler loading, when compared with unfilled XNBR compound, a reduction of volume loss of approximately 49.5%, 49.7%, 51.4%, 58.1%, 55.6% and 51.9% each. As for 35 phr of PKSBc-filled XNBR composite, the volume loss was reduced by 46.2%. This shows that PKSBc helps to prevent the volume loss of rubber from increasing. The volume loss was maintained for all XNBR filled with hybrid CB/PKSBc and PKSBc alone. The porous structure of PKSBc as shown in the SEM image of Figure 2d provided surface area contact for mechanical interlocking between hybrid CB/PKSBc and the XNBR phases which resulted in surface roughness and improved rubber-filler interaction [35]. Hybrid CB/PKSBc reduced the volume loss of XNBR composites hence indicating better abrasion resistance. Therefore, this will contribute to the reduction of wear when the XNBR composite is subjected to any mechanical actions.

#### 3.2.3. Hardness

**Figure 5:**
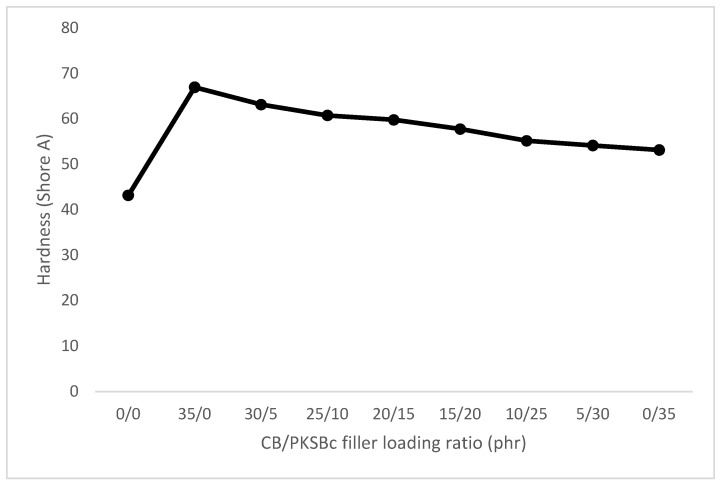
Hardness value of hybrid CB/PKSBc filled XNBR composites.

Figure 5 shows the hardness results for the hybrid CB/PKSBc-filled XNBR composites. Based on Figure 5, the XNBR composite with 0 phr of filler showed the lowest hardness value compared to the other filled XNBR composites. Although there was no reinforcement provided in the composite, it shows a high hardness value, 43.9 Shore A. This is due to XNBR’s nature of being a high-wearing polymer where it has chemical resistance, high abrasion, and high hardness [36]. When 35 phr of CB was added into the XNBR composite, the hardness of the composite was improved to 67 Shore A. CB is a proven reinforcing filler that provides high hardness to the composite, which in this study, improved the hardness of the composite by 35.5%. As displayed in Figure 3, the morphology of CB showed that CB has a high specific surface area and the complex shape of the aggregates helped to provide excellent interfacial contact area with the XNBR matrix [11]. When 35 phr of PKSBc is incorporated, the hardness of the composite was increased to 53.2 Shore A, which indicated an improvement of about 18.8%. Once CB is hybridized with PKSBc at 30/5 phr loading, the hardness of the composite is increased by 31.6%. The high hardness of the XNBR composite filled with 30/5 phr of hybrid CB/PKSBc is due to the high crosslink density of the composite. The pores on the surface of PKSBc promote the formation of crosslink density, hence creating a 3D network that makes the composite stiff and rigid [32]. An increment of PKSBc in hybrid CB/PKSBc causes the hardness of the composite to decrease slightly. This can be seen for the XNBR composites filled with 25/10 phr, 20/15 phr, 15/20 phr, 10/25 phr, and 5/30 phr of hybrid CB/PKSBc, where the composites had a reduction of approximately 3.8%, 5.4%, 8.5%, 12.7% and 14.2% respectively when compared with 30/5 phr of hybrid CB/PKSBc filled XNBR composite. Although with a higher ratio of PKSBc, the hardness of the XNBR composite showed a slight reduction, when compared to the unfilled XNBR, the incorporation of hybrid CB/PKSBc or PKSBc alone enhanced the hardness of the XNBR composite. Based on the SEM image of PKSBc in Figure 2, PKSBc has an irregular shape which improved rubber-filler interaction, resulting in equal relocation of stress concentration from filler to rubber matrix when subjected to external stress [11]. This caused the hardness of the hybrid CB/PKSBc-filled XNBR composite to be maintained at a high value. This shows that PKSBc can be used as a co-filler or be used alone to improve and maintain the hardness of XNBR.

## 4. Conclusions

In conclusion, PKSBc has the potential to be used as a co-filler in rubber reinforcement. PKSBc can be concluded as a diluent filler where it could be hybridized with CB to obtain a final rubber product with high performance. In comparison to raw PKS, PKSBc has oxygen-containing functional groups such as alcohols, phenols, and carboxylic acids. Impurities and lignocellulose components from raw PKS are removed hence creating PKSBc with a porous structure. A higher loading ratio of PKSBc in hybrid CB/PKSBc reduces T_90_ and prolongs the CRI of rubber composites. The incorporation of PKSBc helped to maintain the hardness and volume loss of XNBR which reflects on its ability to provide high hardness and high abrasion resistance to the XNBR matrix. This way, an abundance of palm oil waste, especially palm kernel shell, can be managed by fabricating PKSBc and a more sustainable, yet effective alternative can be carried out to decrease the usage of CB.

## Figures and Tables

**Table 1:** Formulation of hybrid CB/PKSBc filled XNBR composites.

Formulation No.									
Ingredients	1	2	3	4	5	6	7	8	9
XNBR	100	100	100	100	100	100	100	100	100
CB	0	35	30	25	20	15	10	5	0
PKSBc	0	0	5	10	15	20	25	30	35
ZnO	3	3	3	3	3	3	3	3	3
Stearic acid	2	2	2	2	2	2	2	2	2
Accelerator	3.55	3.55	3.55	3.55	3.55	3.55	3.55	3.55	3.55
Sulphur	1.3	1.3	1.3	1.3	1.3	1.3	1.3	1.3	1.3
Processing oil	10	10	10	10	10	10	10	10	10

**Table 2:** Rheological properties of hybrid CB/PKSBc filled XNBR composites.

Hybrid CB/PKSBc Loading (phr)									
Properties	0/0	35/0	30/5	25/10	20/15	15/20	10/25	5/30	0/35
M_L_ (dNm)	2.13	3.12	3.00	3.64	3.63	3.68	3.18	3.08	3.04
M_H_ (dNm)	16.3	24.2	23.4	23.5	25.9	22.2	20.5	23.9	26.5
M_H_ − M_L_ (dNm)	14.2	21.1	20.4	19.9	22.3	18.5	17.3	20.8	23.4
TS2 (min)	2.17	1.58	2.08	2	1.5	2.03	2.11	2.05	2.14
T90 (min)	6.13	5.49	5.54	5.12	4.31	5.1	5.17	4.51	5.5
CRI (min^−1^)	25.3	25.6	28.9	32.1	35.6	32.6	32.7	40.7	29.8

## Data Availability

Not applicable.
